# Dietary fish oil did not prevent sleep deprived rats from a reduction in adipose tissue adiponectin gene expression

**DOI:** 10.1186/1476-511X-7-43

**Published:** 2008-11-05

**Authors:** Ana Barbosa Marcondes de Mattos, Mônica Jordão S Pinto, Cristiane Oliveira, Carolina Biz, Eliane Beraldi Ribeiro, Claudia Maria Oller do Nascimento, Monica Levy Andersen, Sergio Tufik, Lila Missae Oyama

**Affiliations:** 1 Department of Physiology, Federal University of São Paulo, São Paulo, Brazil; 2 Department of Psychobiology, Federal University of São Paulo, São Paulo, Brazil; 3 Department of Bioscience, Federal University of São Paulo, Santos, Brazil; 4 Universidade Federal de São Paulo, UNIFESP, Departamento de Fisiologia Rua Botucatu, 862, 2° andar, Vila Clementino, São Paulo, SP, Brazil

## Abstract

Sleep deprivation in humans has been related to weight gain and consequently, increased risk for insulin resistance. In contrast, there is a significant loss of weight in sleep deprived rats suggesting a state of insulin resistance without obesity interference. Thus, we aimed to assess the effects of a rich fish oil dietetic intervention on glucose tolerance, serum insulin and adiponectin, and adipose tissue gene expression of adiponectin and TNF-α of paradoxically sleep deprived (PSD) rats. The study was performed in thirty day-old male Wistar randomly assigned into two groups: rats fed with control diet (soybean oil as source of fat) and rats fed with a fish oil rich diet. After 45 days of treatment, the animals were submitted to PSD or maintained as home cage control group for 96 h. Body weight and food intake were carefully monitored in all groups. At the end of PSD period, a glucose tolerance test was performed and the total blood and adipose tissues were collected. Serum insulin and adiponectin were analyzed. Adipose tissues were used for RT-PCR to estimate the gene expression of adiponectin and TNF-α. Results showed that although fish oil diet did not exert any effect upon these measurements, PSD induced a reduction in adiponectin gene expression of retroperitoneal adipose tissues, with no change in serum adiponectin concentration or in adiponectin and TNF-α gene expression of epididymal adipose tissue. Thus, the stress induced by sleep deprivation lead to a desbalance of adiponectin gene expression.

## Introduction

The constant search of more time to achieve all daily activities has leaded the major part of world population to deprive some of the sleep hours [1]. The real consequences of this change are still not totally known, but studies have been pointing out for a range of metabolic and hormonal alterations, which unbalance and damage the normal function of the body [2-8]. Nevertless, the population is sleeping 1.5 hours less than a century ago [9]. It seems that this reduction in sleep, which was directly associated with advanced age, has been imposed to the young age with the increase of demand and life style of modern society [10]. Indeed, young people are exposing their health to the development of alterations, which can lead to an increase in the incidence of age-related diseases earlier and with unknown intensity.

Studies indicate that sleep deprivation causes an autonomic desbalance [11,12] with an impairment of the negative feedback control of hypotalamic-pituitary-adrenal axis [13-18,8]. Plasma cortisol is altered over 24 hour of day, predisposing to insulin resistance development [19,2,20]. Furthermore, Spiegel et al [3] proposed that it can be either by direct effect on the components of glucose regulation, or by indirect effect, through the deregulation of appetite, leading to a weight gain and obesity seeing in humans.

Recent prospective studies have shown long term consequences associated with alterations of chronic sleep deprivation, demonstrating a direct correlation between the duration of sleep and Diabetes type 2 incidence [21,9,25]. In fact, sleep deprivation is an environment important factor in the development of insulin resistance (for review see [26]), as well as different types of diets can influence, in terms of improvement or impairment, the insulin sensitivity.

While the wide range of fat acids has been related with the increase in risk for insulin resistance, the long chain polyunsaturated fat acid omega-3, found in large quantity in fish oil, has been pointed as insulin sensitizing, [27-32]. Moreover, although the mechanisms involved are not totally understood, adiponectin seems to have an important role in this positive effect [30,33,31]. Thus, our study aimed to assess the effects of fish oil rich dietetic intervention on glucose tolerance, serum insulin and adiponectin, and adipose tissue gene expression of adiponectin and TNF-α in paradoxically sleep deprived rats.

## Materials and methods

### Animals and treatments

Forty-eight male Wistar rats aged 30 days and weighting 100–150 g were obtained from Federal University of São Paulo Experimental Models Development Center (CEDEME). The rats were housed inside standard polypropylene cages in a temperature-controlled (23 ± 1°C) room with a 12:12 h light-dark cycle (lights on at 07:00 hours). All procedures used in the present study complied with the guidelines established by Ethical and Practical Principles of the Use of Laboratory Animals [34]. This study was approved by Federal University of São Paulo Research Ethics Committee (01419/06).

Rats were fed with rich fish oil (n = 24) or control diet (n = 24) since they were weaned. At 75 days-old, the animals were randomly distributed in 4 different groups: home-cage control with normal diet (CTRL, n = 12); home-cage fish oil diet (CTRL+D, n = 12); paradoxical sleep deprived control fed with normal diet (PSD, n = 12), and paradoxically sleep deprived with fish oil diet (PSD+D, n = 12).

Both diets were prepared according to the recommendations of the American Institute of Nutrition (AIN-93G and AIN-93M) [35], being similar in calories and lipid content (see table 1). The source of lipids for the control diet was soybean oil (Liza, Brazil) and in the fish oil diet was fish oil (Campestre, Brazil). Diet components were casein (Labsynth, Brazil), L-cystine, cornstarch, butylated hydroxytoluene, cellulose and choline bitartrate (Vitafarm, Brazil) and AIN-93 mineral mixture and vitamin mixture (Dyets Inc, USA).

**Table 1:** Experimental diet compositions according to AIN-93G- growth diet and AIN-93M- maintenance diet

**INGREDIENT**	**CONTROL DIET (g/100 g)**		**FISH OIL DIET (g/100 g)**	
	
	**AIN-93 G**	**AIN-93 M**	**AIN-93 G**	**AIN-93 M**
**Casein**	20	14	20	14
**L-cistine**	0.3	0.18	0.3	0.18
**Cornstarch**	62	71.1	62	71.1
**Soybean oil**	8	5	-	-
**Fish oil**	-	-	8	5
**Butylated hydroxytoluene**	0.0014	0.0008	0.0014	0.0008
**Mineral mixture**	3.5	3.5	3.5	3.5
**Vitamin mixture**	1	1	1	1
**Cellulose**	5	5	5	5
**Choline bitartrate**	0.25	0.25	0.25	0.25

### Paradoxical Sleep deprivation

The rats were submitted to single platform PSD platform method, which involved placing the animal on a narrow circular platform (6.5 cm in diameter) placed inside a chamber filled with water to within 1 cm of their upper surface over a period of 96 hours. At the onset of each PS episode, the animal experiences a loss of muscle tone and falls into the water, thus being awakened. The period of 96 h of PSD was chosen since our previous data showed that the most dramatic alterations in lipid [36,37] and hormone [17] concentrations occur at this time point. Food and water were provided ad libitum by placing chow pellets and water bottles on a grid located on top of the chamber. The basis of the feeder was adapted with a plate to avoid pieces of chow falling into the water. The water in the chambers was changed daily throughout the PSD period. Control rats were placed inside the water chamber but, instead of water, the chamber was filled with sawdust. All rats were habituated to their experimental environment for 3 h, 6 h and 12 h, respectively, on the 3 days preceding the onset of the experiment.

### Glucose Tolerance Test

Twelve hours before the end of PSD, the animals were deprived of food for 12 h (10 am to 10 pm). At the 96 h of PSD or equivalent period in control rats, all animals were anesthetized with ketamine/xilazine (66.6/13.3 mg/Kg). The inguinal cavity was exposed and the femoral artery was canulated for the glucose tolerance test. Initially the baseline blood was collected to assess basal glucose and insulin. Then the glucose load (750 mg/Kg of body weight) was administrated and blood was collected after 4, 12, 20, 24, 28 and 32 minutes to measure glucose concentration. The total blood was collected and maintained in a -80°C freezer until assays with ELISA commercial kits for insulin and adiponectin.

### Carcass lipid and protein content

Carcasses were eviscerated, weighed, and stored at -20°C. Lipid content was measured as described by Stansbie et al [38] and standardized using the method described by Oller do Nascimeto and Williamson [39]. Briefly, the eviscerated carcass was autoclaved at 120°C for 90 minutes and homogenized with double the mass of water. Triplicate aliquots of this homogenate were weighed and digested in 3 ml of 30% KOH and 3 ml of ethanol for at least 2 hours at 70°C in capped tubes. After cooling, 2 ml of 12 N H_2_SO_4 _were added and the sample was washed three times with petroleum ether for lipid extraction. Results are expressed as grams of lipid per 100 g of carcass. For protein measurements, aliquots of the same homogenate were heated to 37°C for 1 hour in 0.6 N KOH with constant shaking. After clarification by centrifugation, protein content was measured according to the method described by Lowry et al [40].

### Real Time polymerase chain reaction

Adiponectin and TNF-α mRNA from retroperitoneal adipose tissue (RET) and epididymal adipose tissue (EPI) was quantified by Real Time Polymerase chain reaction (PCR). RNA samples were previously treated with DNAse (DNA-free, Promega, USA). One microgram of each sample was reverse transcribed using an M-MLV Reverse Transcriptase kit (Promega, USA), and cDNA was synthesized in a final volume of 50 μL. The relative level of Adiponectin and TNF-α mRNA was quantified in real time, using SYBR Green primer in an ABI Prism 7700 Sequence detector (both from Applied Biosystems, USA). The relative level of the housekeeping gene hypoxanthine phosphoribosyltransferase (HPRT) was measured. The primers used were: Adiponectin, 5' GAAGTAGACTCTGCTGAGATGG-3' (sense) and 5' TATCAGTGTAGGAGGTCTGTGATG-3' (antisense); TNF-α, 5'CGCTCTTCTGTCTACTGAAC3' (sense) e 5'TTCTCCAGCTGGAAGACTCC3'(antisense) and HPRT, 5'CTCATGGACTGATTATGGACAGGA3' (sense) and 5'GCAGGTCAGCAAAGAACTTATAGC3' (antisense).

Results were obtained using sequence detector Software (Applied Biosystems, USA) and are expressed as a relative increase, using the method of 2^ΔΔCt ^described by Livak and Schmittgen [41].

### Biochemical and Hormonal serum analysis

Serum glucose concentration was measured by an enzymatic colorimetric method using commercial kits (Labtest, Brazil). Serum adiponectin and insulin concentration were quantified using specific enzyme-linked immunosorbent assay (ELISA) kits (Linco Research, USA).

### Statistical Analysis

All results are presented as means ± standard error of the mean (SE). Statistical significances were assessed using two-way analysis of variance (ANOVA) followed by Duncan's test. Differences were considered significant when p < 0.05.

## Results

### Body weight gain and food intake

**Figure 1:**
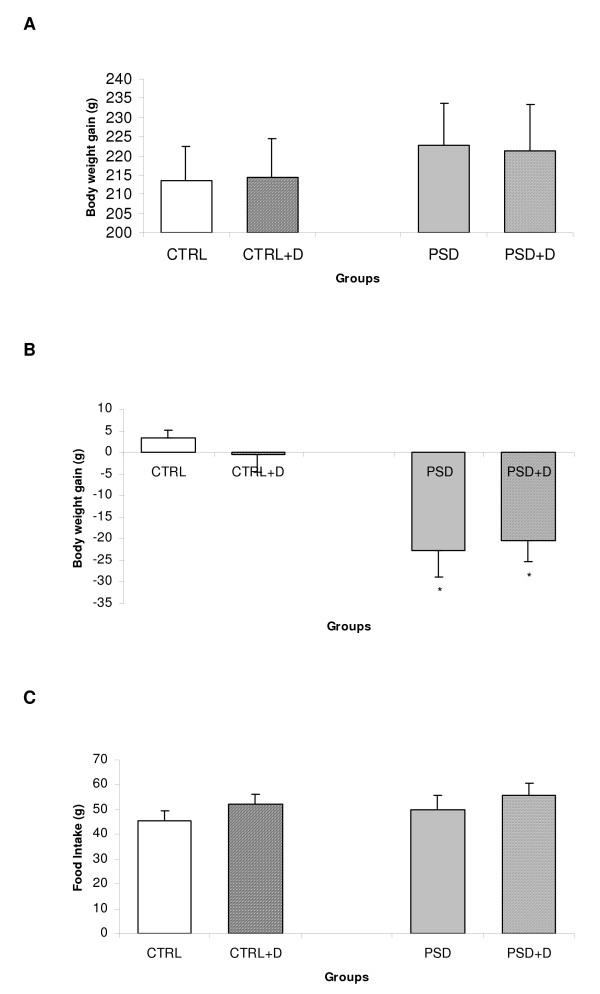
**Alterations in total body weight gain (A) during diet treatment and during paradoxical sleep deprivation (B). **Figure (C) depicts total food intake (g) measured in control (CTRL) and paradoxical sleep deprived (PSD) rats fed with normal or fish oil diet (D). Data are expressed as mean ± SEM. *Different from CTRL rats. N = 10–12 rats/groups.

Body weight and food intake were monitored in all groups of rats throughout the whole experiment period. During the diet treatment period, the total body weight gain was not different between groups (Fig. 1A). After PSD, significant group (CTRL or PSD) effect [*F*_(1,39) _= 36.97; p < 0.001] was revealed by two-way ANOVA. The post-hoc Duncan analysis demonstrated that sleep deprivation decreased body weight in both normal and fish oil diet groups (p < 0.0001), as shown in Fig. 1B. In regards to total food intake, no significant difference was observed between all groups (Fig. 1C).

### Carcass fat content

**Figure 2:**
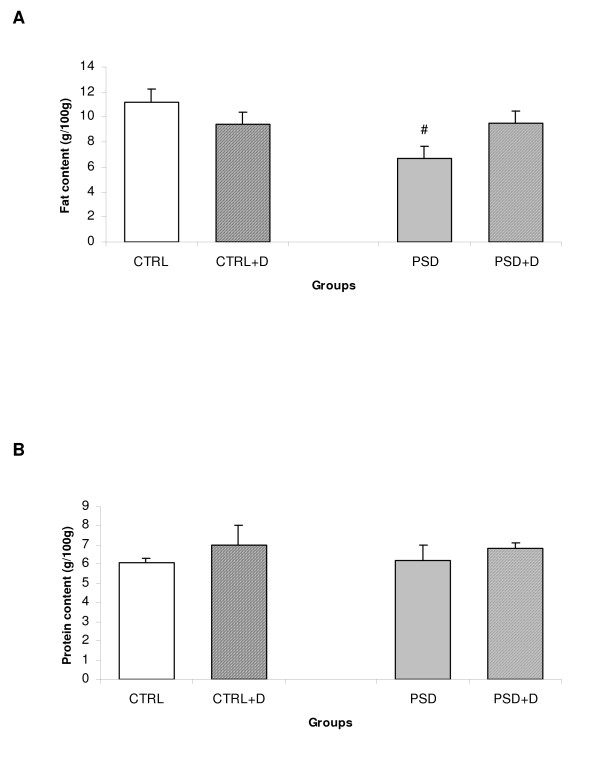
**(A) Carcass lipid content (n = 9 animals/group) and (B) protein content (n = 5 animals/group) in CTRL and PSD rats fed with control or fish oil rich diet.** Data are expressed as mean ± SEM. #Different from all other groups.

The results of carcass fat and protein content are depicted in Fig. 2A. There was a significant interaction [*F*_(1,49) _= 7.52; p < 0.009] between group and diet, in which PSD rats fed with normal diet exhibited significant decreased fat content in the carcass compared with other groups (p < 0.03). No significant difference was observed between groups for protein content (Fig. 2B).

### Glucose Tolerance test

**Figure 3:**
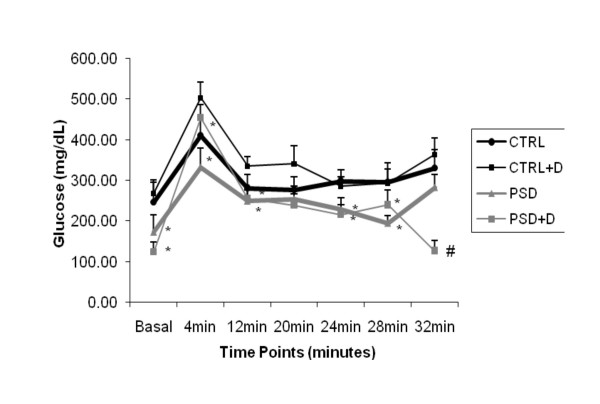
**Glucose tolerance test in CTRL and PSD rats fed with control or fish oil rich diet.** Data are expressed as mean ± SEM. *Different from respective CTRL rats (n = 8–9 animals/group). #Different from all other groups.

The glucose tolerance test showed a group effect [*F*_(1,29) _= 8.31; p < 0.007] during the baseline (p < 0.01), 4° minute (p < 0.005), 12° minute (p < 0.04), 24° minute (p < 0.01), 28° minute (p < 0.03), and 32° minute (p < 0.001). Duncan test analysis demonstrated that the glucose in the CTRL group was significantly higher than in PSD group (p < 0.03), independently of the diet for all these time-points. In the 32° minute of the test, there was also an interaction between group and diet [*F*_(1,33) _= 4.20; p < 0.05]. The Duncan test revealed that the glucose was significantly lower in PSD+D rats than any other group (p < 0.01), as depicted in Fig. 3A.

### Serum, insulin and adiponectin

**Figure 4:**
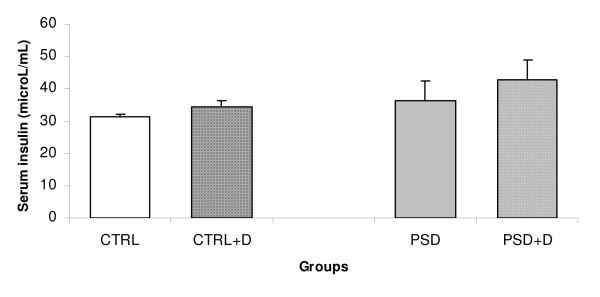
**Serum insulin (n = 5–9 animals/group) in CTRL and PSD rats fed with normal or fish oil rich diet.** Data are expressed as mean ± SEM.

**Figure 5:**
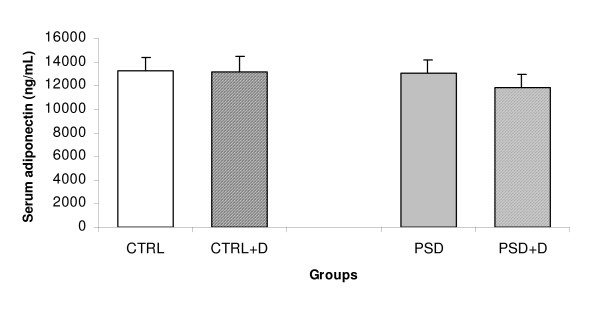
**Serum adiponectin (n = 8 animals/group) in CTRL and PSD rats fed with normal or fish oil rich diet.** Data are expressed as mean ± SEM.

No significant alteration in the serum insulin (Fig. 4) and adiponectin (Fig. 5) was observed in the four groups evaluated.

### Quantification of gene expression

**Figure 6:**
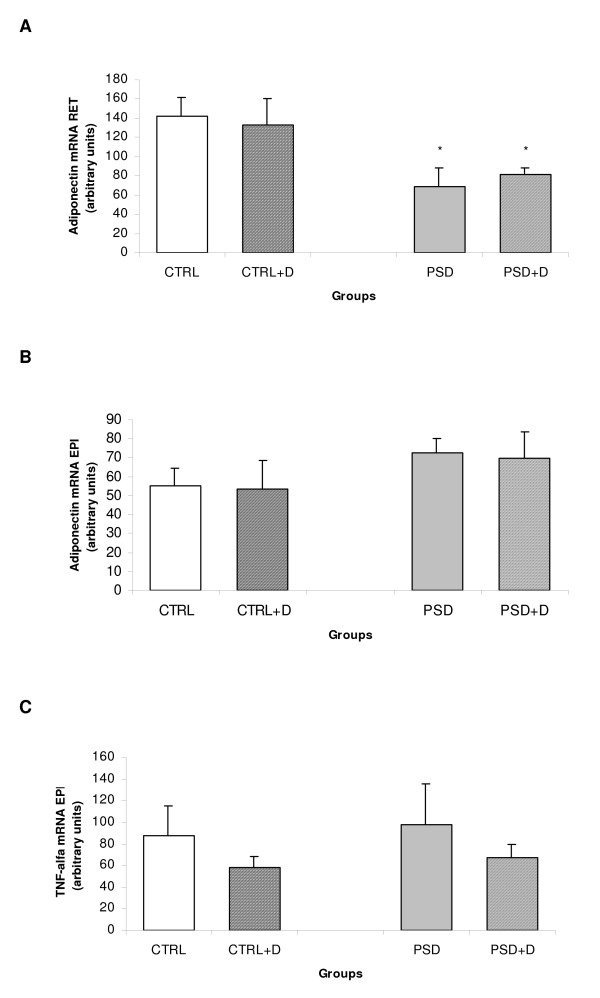
**(A) Retroperitoneal adipose tissue adiponectin mRNA expression (n = 7–8 animals/group); (B) epididymal adipose tissue adiponectin mRNA expression (n = 7–9 animals/group); (C) epididymal adipose tissue TNF-α mRNA expression (n = 8–9 animals/group) in CTRL and PSD rats fed with normal or fish oil diet.** Data are expressed as mean ± SEM. *Different from CTRL rats.

Figure 6 depicts adiponectin and TNF-α gene expression in adipose tissue of all groups. ANOVA revealed a significant group effect [*F*_(1,26) _= 9.53; p < 0.004]. The Duncan test showed that adiponectin gene expression in retroperitoneal adipose tissue was significantly lower in PSD rats compared with CTRL rats (p < 0.004), as shown in Fig. 6A. Further, adiponectin and TNF-α gene expression was not different between all groups in epididymal adipose tissue (Fig. 6B) (Fig.6C).

## Discussion

In the present study we investigated the effect of a fish oil diet prior and during the period of 96 hours of PSD in metabolic parameters involved in insulin resistance of rats. As previously described, sleep deprivation induced a significant loss of weight [42-48] and increased corticosterone concentrations [49,16,17] after 96 h of PSD protocol, being related to an increased energy expenditure and thus, a negative energy balance. The carcass fat content was lower in sleep deprived rats, as previously reported by Hipolide et al. (2006) while the protein carcass content was not different between groups.

In contrast with previous studies, we found no difference in total food intake. As in our protocol we included an apparatus under the feeders, it may have avoided the waste mentioned recently [48,50]. For instance, these authors reported that there was not an increase in food intake in sleep deprived rats, but a stereotyped gnawing behavior that overestimates the food intake as far as they do not have access to food crumps in the platform technique.

Fish oil has been associated to preventing weight loss during severe physical stress [51,52]. However, results herein showed that long-term fish oil diet did not alter body weight. The reasons for these discrepancies may be attributed to the specific anti-catabolic effect of fish oil in preserving loss in protein content that did not occur in the sleep deprived rats. Nevertheless, Papaconstantinou et al [53] reported no difference in body weight of control rats but a larger reduction for fish oil fed rats, exaggerating the sleep deprivation stress-induced weight loss. In this sense, the high fat diet used in this study could act as an extra stressor effect leading to a marked loss of weight. Further, high fat diet increased TNF-α gene expression in adipose tissue of rats [54]. Collectively, high fat diet predispose for insulin resistance, even the source of fat [55,56], suggesting that fish oil can be also harmful if offered in high doses.

To surrogate indexes for insulin sensitivity/resistance we measured the insulin and glucose concentrations under 12 hours of fasting condition and also after a glucose load. We found a higher glycemia, as well as glucose impaired in non sleep deprived rats when compared to PSD rats. A glucose tolerance test without previous anesthesia was performed after repeated restrain and showed no difference in serum concentrations of glucose, while the insulin was higher when compared to the control [57]. A determinant limitation of the current study is that glucose tolerance test was performed in anesthetized animals. Since all groups have been under the same procedure, the biological effect may be attributed to direct effect of sleep loss on serum analysis. Of note, future studies should be conducted in order to address this issue in unanesthetized rats. Recently, using multiple modified platform technique, Hipolide et al [47] found lower insulin levels in PSD male rats. Thus, insulin levels may also being influenced by other external stressors events.

The major finding of the present study was the reduction in adiponectin retroperitoneal adipose tissue gene expression in PSD rats when compared to control rats, not accompanied by alterations in epididymal adipose tissue for adiponectin and TNF-α and/or serum adiponectin levels. In addition, the fish oil diet did not prevent this impaired effect induced by sleep deprivation. These data suggest that the fat content in normolidic diet was not sufficient to regulate adiponectin expression or maybe the period of treatment of 45 days was not long enough to result in improvement. Pighin et al [29] reversed the whole body peripheral insulin sensitivity with 60 days of a normolipidic fish oil in insulin resistance rats.

Adiponectin expression and serum levels decrease with obesity and are positively associated with weigh loss [58]. In contrast, Yamauchi et al [59] showed that adiponectin is decreased in lipoatrophic rats. Thus, its gene expression regulation remains unclear, but studies have shown that TNF-α and glucocorticoids decrease adiponectin gene transcription [60-62].

The dynamics of circulating adiponectin was analyzed in humans showing that the 24 h serum adiponectin variations followed those of cortisol with a 2 h lag period later during the day and at night [63]. These authors hypothesized that the nocturnal cortisol decline, indirectly determines compensatory adiponectin changes that would tend to keep the degree of insulin resistance stable. The results maybe explain why the serum adiponectin did not have the same reduction of gene expression in PSD rats, and suggest that more time would be necessary for changings in dynamic variation of cortisol rather to provoke direct action in serum adiponectin. Furthermore, another study focusing fasting state, had no difference in serum adiponectin while the gene expression was reduced, supposing that the large concentration in blood may serve to buffer this hormone against any sudden rise or fall in response to an acute internal or external signal [64].

Interestingly, when analyzing to the comparative physiology of these systems to humans, there is a main important effect of sleep deprivation on adiponectin gene expression programming besides the presence of obesity. Thus, our data showed, as a whole, that sleep deprivation leads to a significant decrease in adiponectin gene expression, which cannot be prevented by a rich fish oil diet. It seems that the stress induced by PSD leads to an impairment in adiponectin gene expression function of retroperitoneal adipose tissue, showing a specific insulin sensitivity negative impact on an adipose tissue depot, independently of weight loss seen in PSD rats.

## Competing interests

The authors declare they have no competing interests.

## Authors' contributions

ABMM have made substantial contributions to conception and design, all the experimental analysis and acquisition of data and also analysis and interpretation of data. MJSP carried out the immunoassays and in the insulin receptor analyze. CO participated in all molecular and biochemical analyzes. CB participated in all molecular and biochemical analyzes. EBR participated in the design of the study and helped to draft the manuscript. CMON participated in the design of the study and performed the statistical analysis. MLA participated in the design of the study and performed the statistical analysis and helped to draft the manuscript. ST participated in the design of the study and performed the statistical analysis and helped to draft the manuscript. LMO has made substantial contributions to conception and design, analysis and interpretation of data and coordination to draft the manuscript. 
